# The Corrective Role of Melatonin in Synergism of Dark Deprivation and CCl_4_ Intoxication in the Pathogenesis of Liver Damage a in Rats

**DOI:** 10.3390/cimb47121046

**Published:** 2025-12-15

**Authors:** Sevil A. Grabeklis, Liudmila M. Mikhaleva, Alexander M. Dygai, Rositsa A. Vandysheva, Anna I. Anurkina, Maria A. Kozlova, David A. Areshidze

**Affiliations:** 1 Avtsyn Research Institute of Human Morphology of Petrovsky National Research Centre of Surgery, 117418 Moscow, Russia; 2 Research Institute of General Pathology and Pathophysiology, 125315 Moscow, Russia

**Keywords:** circadian rhythms, dark deprivation, carbon tetrachloride, melatonin, hepatoprotection, desynchronosis, liver

## Abstract

Circadian rhythm disruption induced by exposure to light—excessive in duration and intensity (dark deprivation)—and the impact of hepatotoxins are both significant risk factors for liver pathology. The purpose of this research was to evaluate the potentially synergistic effects of continuous lighting and carbon tetrachloride (CCl_4_) toxicity on the structural and functional organization and daily (circadian) rhythmicity of the liver in rats, as well as to look at the corrective capability of exogenous melatonin under such influences. The experiment was conducted on 200 outbred 6-month-old Wistar rat males, which were distributed into five groups, including a control (normal light/dark cycle), dark deprivation (constant light), CCl_4_ intoxication, and combined exposure to CCl_4_ and dark deprivation with or without melatonin administration (0.3 mg/kg). Histological, immunohistochemical (Ki-67, Per2, and Bmal1), biochemical, and ELISA methods were used. Circadian rhythms were analyzed using cosinor. It was shown that dark deprivation and CCl_4_ intoxication act synergistically, potentiating liver damage. The most severe necrosis (54.17 ± 9.13%), steatosis (57.85 ± 12.14%), and suppression of regenerative potential (decreased proportion of binucleated hepatocytes to 2.17 ± 0.21%) were observed in the group with combined exposure. This correlated with a substantial decline in melatonin content in blood plasma (7.85 ± 2.1 pg/mL) and a profound disruption in circadian rhythms. Administration of exogenous melatonin exerted pronounced hepatoprotective and chronotropic effects: it significantly reduced pathological changes (necrosis reduced to 16.35 ± 6.17%), stimulated regeneration (binucleated hepatocytes increased to 13.57 ± 0.81%), and restored the circadian rhythms of the studied parameters to levels close to those of the control. The key pathogenetic link in the potentiation of CCl_4_ hepatotoxicity under dark deprivation is light-induced deficiency of endogenous melatonin. Exogenous melatonin demonstrated high efficacy in correcting both structural and functional damage and liver desynchronosis, confirming its therapeutic potential under conditions of combined exposure to chronodisruptors and toxins.

## 1. Introduction

Carbon tetrachloride or tetrachloromethane (CCl_4_, TCM) is a hepatotoxic agent [1] widely used in experimental research to study liver damage and recovery. CCl_4_-induced toxicity refers to the multiple toxic effects of CCl_4_, which depend primarily on the method of peripheral administration, dose, and duration of exposure. Hepatotoxic effects are those that cause negative changes in liver cell homeostasis with pronounced morphological and functional manifestations.

The complex of changes taking place in the liver during exposure to TCM includes morphological and functional alterations in hepatocytes and their organelles, primarily mitochondria, and manifests itself as changes in the functional biochemical constants characterizing liver function. Data on the relationship between the toxic influence of CCl_4_ and changes in biorhythms are limited, and the overwhelming majority of studies examine the response of an organism as a whole—and the liver in particular—under the influence of TCM during various rhythm’s periods, rather than the effect of this active substance on circadian rhythms [2].

Thus, although morphofunctional changes in the liver induced by carbon tetrachloride have been described in sufficient detail, a key aspect of their dynamics and, possibly, the mechanism of their development remains insufficiently understood. Given the fundamental role of circadian rhythms in regulating hepatocyte metabolism and the body’s ability to adapt to stress, the question naturally arises: how exactly does carbon tetrachloride exposure affect not only the liver’s condition, but also the internal temporal organization of its functions? Research into this question requires a shift in focus from studying the organ’s response at a single time point to analyzing how toxic exposure disrupts the coordinated circadian rhythms of biochemical and cellular processes underlying liver homeostasis.

The circadian rhythmicity of biological functions is the basic property of any living organism. These rhythms represent oscillations of an endogenous nature, the period of which approaches 24 h. These fluctuations allow organisms to predict daily environmental patterns and adjust their reactions depending on the time of day [3]. Thus, the existence of circadian rhythmicity ensures adaptation to existence under the light cycle [4,5]. Maintenance of the circadian rhythm of the body, both normally and under the effect of a variety of endo- and exogenous factors, is regulated by a complex system involving central and peripheral components, as well as an intracellular “clock”. The activity of this “clock” is particularly noticeable in cells with high metabolism, such as hepatocytes [6,7].

For the liver, it has been shown that the rhythmicity of vital processes in this organ is highly dependent on changes in the external environment. The endogenous nature of circadian oscillations in the liver, first described in 1998 [7,8], is currently considered to be more stable than in other organs, and at the same time possesses a number of organ-specific features [5,9]. Disruption of structure of circadian rhythms of liver serves as a basis for development of various pathological conditions and accelerated aging [5,10,11,12,13].

In modern conditions, the leading factor causing disruption of the normal daily rhythm of liver function is exposure to light at night, most often of anthropogenic origin [14,15].

The development of desynchronosis—preceding more unfavorable morphofunctional changes in organs in the case of lengthening the light part of the day—causes suppression of the melatonin-synthesizing function of the pineal gland, as well as disruption of the CR of melatonin production, which leads to significant morphofunctional changes in the liver [16,17,18]. Thus, when the lighting regime is disrupted, the liver is exposed to adverse effects due to a deficiency of pineal melatonin on the one hand, and due to the development of desynchronosis on the other.

The antioxidant effect of melatonin is responsible for its hepatoprotective effect, which has been described in a number of studies [19,20,21].

Our previous studies have shown that the combination of CCl_4_ exposure and prolonged dark deprivation results in more significant disturbances in the structure and function of hepatocytes and also leads to significant changes in the circadian rhythms of several parameters, which were not observed with CCl_4_ as a single factor. We hypothesize that a key factor determining the severity of these structural and functional changes is light-induced suppression of synthesis and secretion of pineal melatonin, which normally provides a hepatoprotective effect [21].

However, it is unknown to what extent melatonin deficiency causes CCl_4_ hepatotoxicity, how effective the use of exogenous melatonin will be as a hepatoprotector in toxic liver damage, and what impact it will have on the circadian rhythms associated with the structural and functional integrity of the liver.

The aim of this study was to determine whether dark deprivation (constant light) exacerbates CCl_4_-induced hepatotoxicity by suppressing endogenous melatonin production and to assess whether exogenous melatonin could mitigate this combined damage and the associated circadian rhythm disruption (desynchronosis).

## 2. Materials and Methods

### 2.1. Study Subjects

The experiment was performed using 200 male outbred Wistar rats aged 6 months, with an average body weight of 300.0 ± 35.8 g.

Animals were obtained from the Laboratory Animal Nursery “Pushchino” of the Shemyakin–Ovchinnikov Institute of Bioorganic Chemistry, Russian Academy of Sciences (IBCh RAS).

All animals were kept in plastic cages at a temperature of 20–22 °C and a relative humidity of 60–70%. Rats had free access to drinking water and briquetted feed. The animals were given tap water that complied with Russian sanitary standards for drinking (SanPiN 2.1.4.1074-01—sanitary and epidemiological rules and regulations “Drinking Water. Hygienic Requirements for the Quality of Water in Centralized Drinking Water Supply Systems. Quality Control Resolution of the Chief State Sanitary Doctor of the Russian Federation”). Drinking bowls were checked regularly to ensure proper maintenance, cleanliness, and functionality. The source of feed was briquetted feed PK120-1 (OOO Laboratorsnab, certificate of conformity No. POCCRU.nO81.B00113, GOST P50258-92). Animal keeping and experiments were performed in accordance with the European Convention for the Protection of Vertebrate Animals used for Experimental and other Scientific Purposes (Strasbourg, 18 March 1986). Permission for this study was obtained from the Bioethics Committee of Avtsyn Research Institute of Human Morphology.

Animal inclusion criteria for the experiment were as follows: body weight 350 ± 40 g, absence of external defects, injuries, or behavioral abnormalities. The exclusion criterion was the death of an animal during the experiment, but no such cases were observed.

### 2.2. Study Design

Animals were randomly divided into five groups (n = 40 per group).

The control group (n = 40) was maintained on a 12:12 h light–dark cycle (lights on at 08:00).

Group I (n = 40) was subjected to constant light (24 h dark deprivation).

Group II (n = 40) was kept under control conditions but treated with intraperitoneal CCl_4_ (Sigma-Aldrich, Cat# 289116, St. Louis, MO, USA) (in olive oil (Sigma-Aldrich, Cat# O1514, St. Louis, MO, USA); 0.2 mL/100 g of body weight) every three days. The treatment regimen consisted of seven injections per rat [22].

Group III (n = 40) was maintained under dark-deprivation conditions and also received CCl_4_ injections as described above.

Group IV (n = 40) was maintained under the same conditions as Group III but received a daily intragastric administration of melatonin aqueous solution (Sigma-Aldrich, Cat# M5250, St. Louis, MO, USA) at a dose of 0.3 mg/kg of body weight [23]. Administration was performed in the evening (between 8:00 p.m. and 9:00 p.m.) to ensure that the peak blood melatonin concentration occurred at night.

At the three-week endpoint, rats were euthanized via guillotine decapitation. Euthanasia was staggered across a 24 h cycle (09:00, 15:00, 21:00, 03:00) to capture diurnal rhythms, with 10 animals sacrificed per time point. Before euthanasia, rectal temperature was recorded, and blood samples were drawn for hematological and biochemical analyses. A complete necropsy followed.

The number of animals per group was determined to guarantee the statistical reliability of the cosinor-based circadian rhythm analysis. A sufficient group size is necessary to minimize the confounding effects of individual animal differences. Based on the established literature, a sample size of n = 10 per group provides approximately 80% statistical power at a significance level of α = 0.05; therefore, we used 10 animals per time point—resulting in 40 animals per experimental group—to ensure reliable results [24,25,26].

Decapitation was chosen over chemical euthanasia to avoid confounding effects on biochemical and histological parameters. Chemical agents can alter blood plasma composition (e.g., CO_2_ induces acidosis; KCl precludes potassium analysis) and compromise tissue integrity, as seen with intraperitoneal pentobarbital or alcohol, which diminishes tinctorial quality in histological sections [27]. Accurate chronobiological analysis requires that euthanasia at each time point be completed within a narrow interval to minimize temporal variance. Decapitation is the most suitable method to achieve this rapid and synchronized tissue collection.

At least 4 preparations from one animal were analyzed, with at least 10 fields of view. For each animal, several investigators were involved, but only the first investigator, who administered CCl_4_ and melatonin, knew about the animals’ group assignments. Euthanasia was performed by a different investigator, and all other studies were conducted by investigators blinded to the animals’ group assignments.

### 2.3. Morphological, Morphometric, and Histochemical Study Methods

Liver samples from the left lobe were fixed in 10% neutral buffered formalin (BioVitrum, Cat# F-002, St. Petersburg, Russia), dehydrated through a graded ethanol series, cleared in xylol, and embedded in Histomix histological medium (BioVitrum, Cat# H-103, St. Petersburg, Russia). Serial sections (5–6 μm) were cut using a Leica SM2010 R rotary microtome (Leica Biosystems, Nussloch, Germany) and stained with hematoxylin and eosin (H&E) (BioVitrum, Cat# HE-101, St. Petersburg, Russia) following standard protocols.

To confirm the presence of fatty degeneration in hepatocytes, serial frozen liver sections (6–8 μm) were cut with use of a cryostat table for the “Unicon” MFT-01 microtome (Unicon, Moscow, Russia) and stained with Sudan-III (Sigma-Aldrich, Cat# 861340, St. Louis, MO, USA) in 70% ethanol.

Digital images were acquired from histological sections using a Leica DM 2500 (Leica SM2010 R, Leica Biosystems, Wetzlar, Germany ) microscope and a DFC 290 camera (Leica SM2010 R, Leica Biosystems, Wetzlar, Germany). From each sample, ten randomly selected fields were photographed at 400× and 1000× magnification for later karyometric and cytometric analyses [28].

Using Qupath (Edinburgh, UK) 0.5.1 software with relevant plugins [29], the cross-sectional areas of the nuclei (Sn) and cytoplasm (Sc) were measured in micrometers. The software was geometrically calibrated using an object-micrometer slide digitized at the same magnification. Analyses were restricted to morphologically normal hepatocytes, and the proportion of binucleated cells was also quantified.

The percentage of necrotic hepatocytes relative to the total number of hepatocytes in the field of view was determined.

The nuclear–cytoplasmic ratio was determined according to the following formula [30]:

NCR = S_n_/S_c_

### 2.4. Immunohistochemical Methods

For immunohistochemical (IHC) staining, liver sections were dewaxed, rehydrated, and treated with 3% hydrogen peroxide (PanReac AppliChem, Cat# 131339, Darmstadt, Germany) to block endogenous peroxidase activity. Following antigen retrieval by heating in citrate buffer (pH 6.0) (Abcam, Cat# ab93678, Cambridge, UK), the sections were incubated with Ultra V Block (Thermo Fisher Scientific). Subsequently, the sections were incubated with primary antibodies.

The following antibodies were used:

Ki-67—Rabbit polyclonal (Cloud-Clone Corp., Cat# PAB063Ra01, Katy, TX, USA), 1:300);

Per2—Rabbit polyclonal (Cloud-Clone Corp., Cat# PAB463Ra01, Katy, TX, USA), 1:200);

Bmal1—Rabbit polyclonal (Cloud-Clone Corp., Cat# PAB461Ra01, Katy, TX, USA), 1:200).

Following a 60 min incubation with primary antibodies at room temperature, binding was detected using the UltraVision Quanto Detection System (Thermo Fisher Scientific, Cat# TL-125-QHL, Waltham, MA, USA).

Control reactions were performed by replacing the primary antibodies with phosphate-buffered saline (PBS) (Sigma-Aldrich, Cat# P4417, St. Louis, MO, USA).

After processing, the slides were dehydrated, cleared in xylene, and coverslipped using BioMount mounting medium (BioVitrum, Russia).

IHC results were quantified by determining the percentage of immunopositive nuclei per total number of hepatocytes, based on the observed nuclear localization of the target antigen. The analysis was performed by counting stained and total cells in four random fields of view at 400× magnification, and the index was calculated as (stained cells/total cells) × 100%.

Two investigators independently performed all morphological, morphometric, histochemical, and IHC studies.

### 2.5. Biochemical Methods

The plasma concentrations of total protein, albumin, alanine aminotransferase (ALT), aspartate aminotransferase (AST), and glucose were measured in rat blood plasma using a StatFax-3300 analyzer (Awareness Technology Inc., Palm City, FL, USA) with the corresponding Spinreact kits (Spinreact, Girona, Spain):

- Total Protein Kit: Spinreact, Cat# 41021, Girona, Spain.
- Albumin Kit: Spinreact, Cat# 41011, Girona, Spain.
- ALT (Alanine Aminotransferase) Kit: Spinreact, Cat# 41211, Girona, Spain.
- AST (Aspartate Aminotransferase) Kit: Spinreact, Cat# 41221, Girona, Spain.
- Glucose Kit: Spinreact, Cat# 41013, Girona, Spain.

### 2.6. Immunoassay Methods

The quantitative determination of plasma melatonin levels was performed via enzyme immunoassay on a StatFax 4200 analyzer (Awareness Technology Inc., Palm City, FL, USA) using a specific ELISA kit from Cloud-Clone Corp (Cloud-Clone Corp., Cat# CEA981Ge, Katy, TX, USA).

### 2.7. Methods for Statistical Processing

Data are presented as mean ± standard deviation (SD). The sample size of 40 represents the number of biological replicates (animals) for each analysis, as specified in the figure legends and table footnotes. For all comparisons of mean daily values between groups, normality was assessed using the Shapiro–Wilk test, and homogeneity of variances was checked with the Brown–Forsythe test. For data satisfying both assumptions, one-way ANOVA was performed, followed by Tukey’s post hoc test for multiple comparisons. For data that violated the assumption of normality or equal variances, the non-parametric Kruskal–Wallis test was used, followed by Dunn’s post hoc test for multiple comparisons. A *p*-value < 0.05 was defined as the threshold for statistical significance. All analyses were conducted in GraphPad Prism, version 6.0. (GraphPad Software Inc., San Diego, CA, USA).

The amplitude and acrophase of circadian rhythms for the studied parameters were determined using cosinor analysis with the CosinorEllipse2006-1.1 program (CosinorEllipse, Version 2006-1.1, Moscow, Russia) [31,32].

Hepatic steatosis was quantified using the NAFLD Activity Score (NAS) protocol. The steatosis score was assigned based on the percentage of affected hepatocytes: 0 for <5%; 1 for 5–33%; 2 for 34–66%; and 3 for >66%. Necrosis was scored on a scale of 0 to 3 as follows: 0, none; 1, 1–5%; 2, 6–20%; 3, ≥20% [33].

## 3. Results

### 3.1. Enzyme Immunoassay Studies

**Figure 1:**
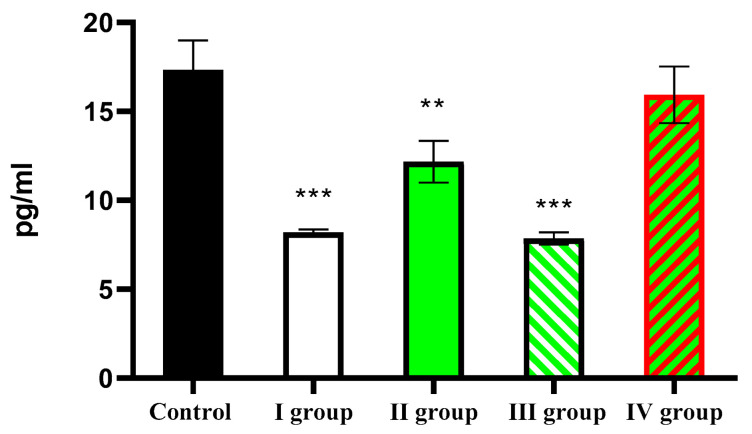
Average daily blood melatonin levels in rats. Note: hereinafter, ** *p* ≤ 0.005, *** *p* ≤ 0.0005 vs. control group.

This study revealed that the average daily melatonin content in the blood of intact animals was 17.34 ± 1.64 pg/mL. Under dark-deprivation conditions, this parameter significantly decreased to only 8.19 ± 1.20 pg/mL. Administration of CCl_4_ also led to a significant reduction in hormone levels, but this effect was not as strong as that caused by dark deprivation. In Group II of rats, the average daily hormone concentration in the blood was 12.17 ± 1.19 pg/mL. The combination of CCl_4_ and constant illumination also resulted in low melatonin levels—7.85 ± 2.1 pg/mL. In rats of Group IV, the same value was 15.94 ± 1.59 pg/mL (Figure 1).

### 3.2. Morphological Studies

**Figure 2:**
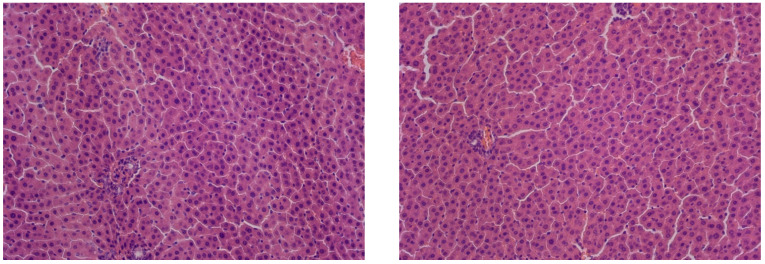
Liver of control animals, hematoxylin and eosin, -×200.

The control group exhibited age-appropriate liver morphology. The liver tissue showed intact trabeculae, consisting of polygonal hepatocytes, containing rounded, centrally positioned nuclei. No pathological changes or necrosis were observed in the hepatocytes of this group (Figure 2).

**Figure 3:**
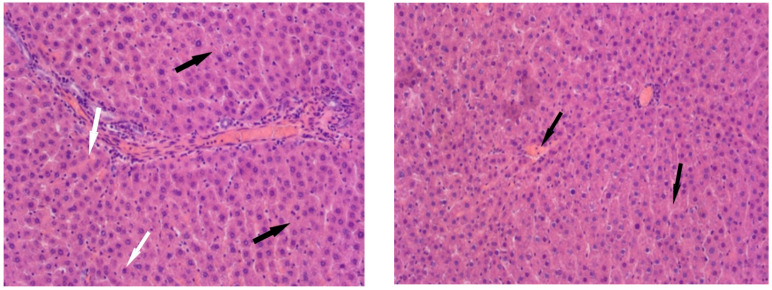
Liver of animals of Group I, hematoxylin and eosin, ×200. White arrows: small-droplet fatty degeneration; black arrows: single necrotic hepatocytes.

In rats kept under constant illumination, no significant differences in liver structure were observed compared to controls. While the overall liver architecture was largely preserved, we observed isolated necrotic hepatocytes alongside cells exhibiting small-droplet fatty degeneration (Figure 3).

**Figure 4:**
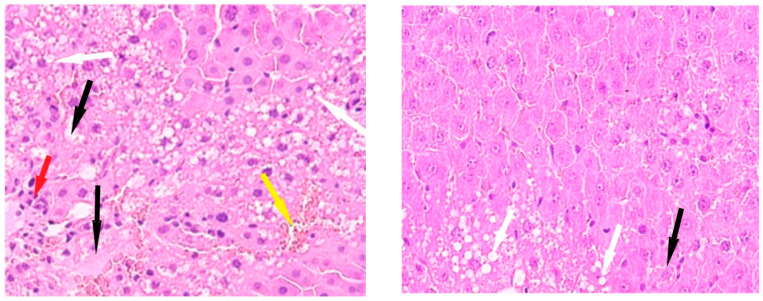
Liver of animals of Group II, hematoxylin and eosin, -×400. White arrows—fatty degeneration; black arrows—necrotic hepatocytes; red arrow—lymphocyte infiltration; yellow arrow—erythrocyte lysis.

In the animals of Group II, the liver structure undergoes significant changes. In the partially preserved organ, liver lobules are visible, and a significant portion of the hepatocytes show no signs of pathological changes. However, some hepatocytes exhibit small- and large-balloon fatty degeneration. In addition, both isolated hepatocytes and foci of necrosis are observed. Lymphocyte infiltration is observed near the liver tracts, and erythrocyte lysis is observed in the capillaries (Figure 4).

**Figure 5:**
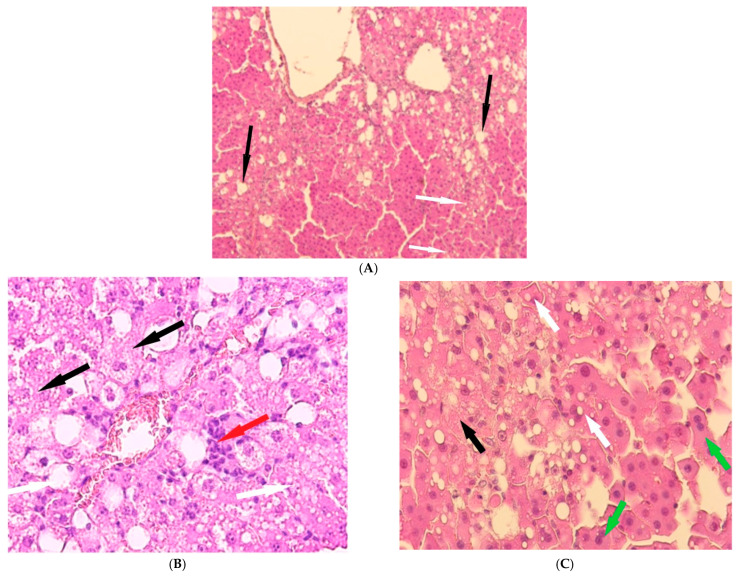
Liver of animals of Group III, hematoxylin and eosin: (**A**) -×100; (**B**,**C**) -×400. White arrows—fatty degeneration; black arrows—necrotic hepatocytes; red arrow—lymphocyte infiltration; green arrow—binucleated hepatocytes.

Histological examination of Group III revealed architectural disruption of the liver trabeculae accompanied by extensive large-droplet fatty degeneration of hepatocytes; centrilobular necrosis of hepatocytes is observed; significant foci of necrosis are also found (Figure 5).

**Figure 6:**
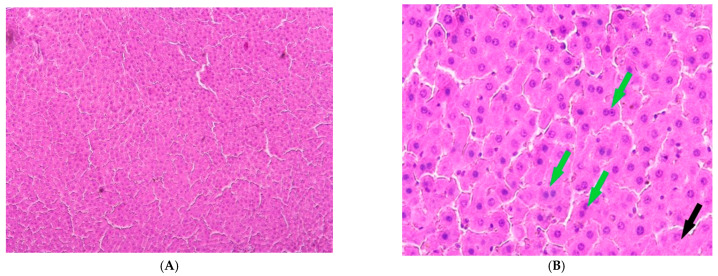
Liver of animals of Group IV, hematoxylin and eosin: (**A**) -×100; (**B**) -×400. Black arrows—necrotic hepatocytes; green arrow—binucleated hepatocytes.

The livers of Group IV animals exhibited preserved liver trabeculae, along with isolated necrotic hepatocytes and scattered fatty degeneration (Figure 6).

The control group exhibited low values for all damage indicators, which is typical of a normal, healthy liver: a minimal NAS index, a low proportion of hepatocytes with lipid droplets, and a low proportion of necrotic hepatocytes. The proportion of binucleated hepatocytes (7.04%) was at a physiological level, indicating a normal level of regenerative processes. Under conditions of dark deprivation, an increase in NAS and lipid accumulation, along with an increased count of necrotic hepatocytes, was observed.

CCl_4_ exposure resulted in more severe pathological changes than in the previous group. However, the proportion of binucleated hepatocytes (5.14%) is no different from the control, which may indicate suppression of the organ’s regenerative potential due to extensive liver damage.

Group III presents the most paradoxical picture. On the one hand, the NAS index and steatosis values reach critical levels, while the proportion of necrosis remains extremely high, and the proportion of binucleated hepatocytes decreases sharply compared to all other groups; this may indicate complete exhaustion of the liver’s regenerative capacity.

Melatonin use results in improvements in all key parameters compared to the other groups of the experiment (Table 1).

The effects of dark deprivation were an enlargement in the hepatocyte cross-sectional area and a decrease in nucleocytoplasmic ratio (NCR). Both CCl_4_ alone and in combination with dark deprivation resulted in an increase in hepatocyte cross-sectional area and a reduction in cross-sectional area of nuclei and the NCR. Similar parameters in hepatocytes of rats in the Group IV (combined exposure to dark deprivation, carbon tetrachloride, and melatonin) showed no statistically significant difference from the control group values (Table 2).

### 3.3. Immunohistochemical Studies

**Figure 7:**
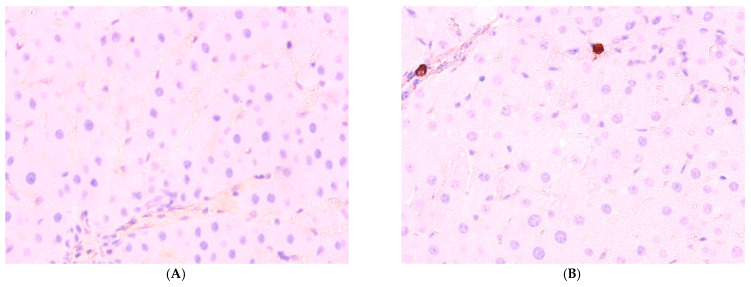

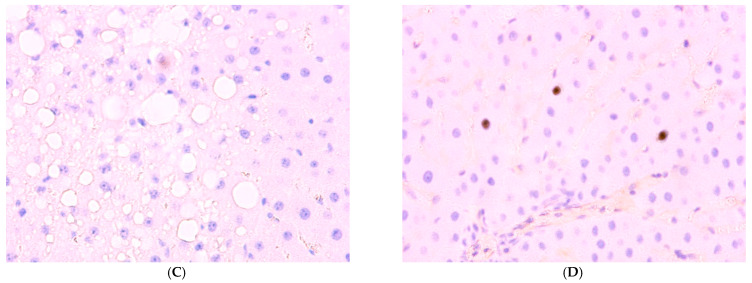
Immunohistochemical reaction to Ki-67 protein in rat liver: (**A**) control, (**B**) Group II, (**C**) Group III, (**D**) Group IV, ×400.

The percentage of *Ki-67*-positive hepatocytes in the liver of control animals is 1.1 ± 0.21%; in Group I (dark deprivation), the proportion increased slightly; in Group II (CCl_4_ exposure), a more substantial increase to 8.14 ± 1.17% was observed. The combined effect of dark deprivation and CCl_4_ (Group III) did not cause significant changes in this indicator. In contrast, in Group IV (dark deprivation, CCl_4_, and exogenous melatonin), a significant increase to 6.35 ± 1.15% was observed (Figure 7).

**Figure 8:**
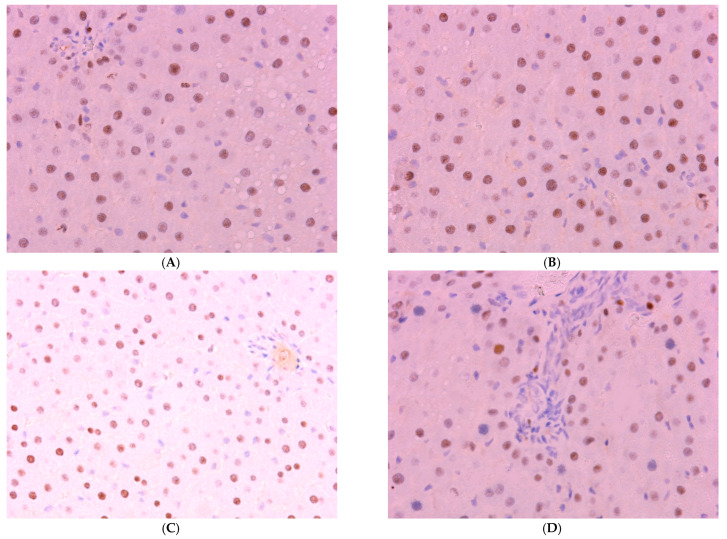
Immunohistochemical reaction for Per-2 protein in rat liver: (**A**) control, (**B**) Group II, (**C**) Group III, (**D**) Group IV, ×400.

The proportion of *Per2*-expressing hepatocytes in the control is 34.51 ± 5.17%; in the first two experimental groups, it increases to 44.51 ± 6.35% and 55.56 ± 8.14%, respectively; in Group III, its level in the liver decreases to 21.57 ± 6.80%; in Group IV rats, it shows no significant difference from control values (Figure 8).

**Figure 9:**
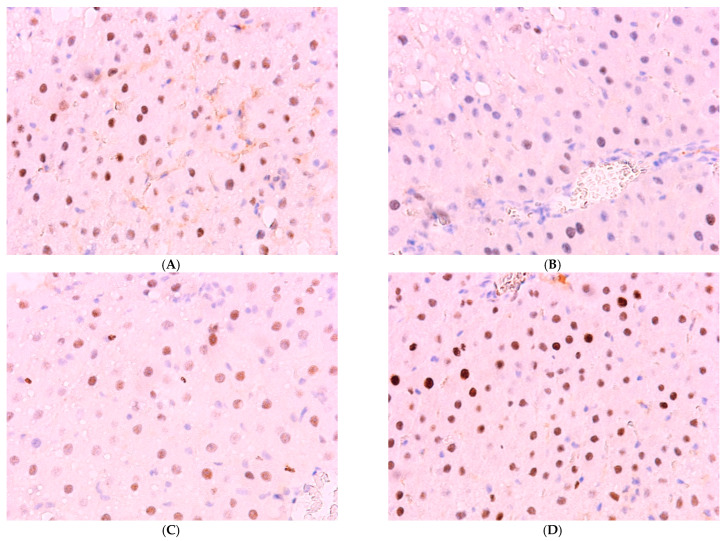
Immunohistochemical reaction for *Bmal-1* protein in rat liver: (**A**) control, (**B**) Group I, (**C**) Group III, (**D**) Group IV, ×400.

**Figure 10:**
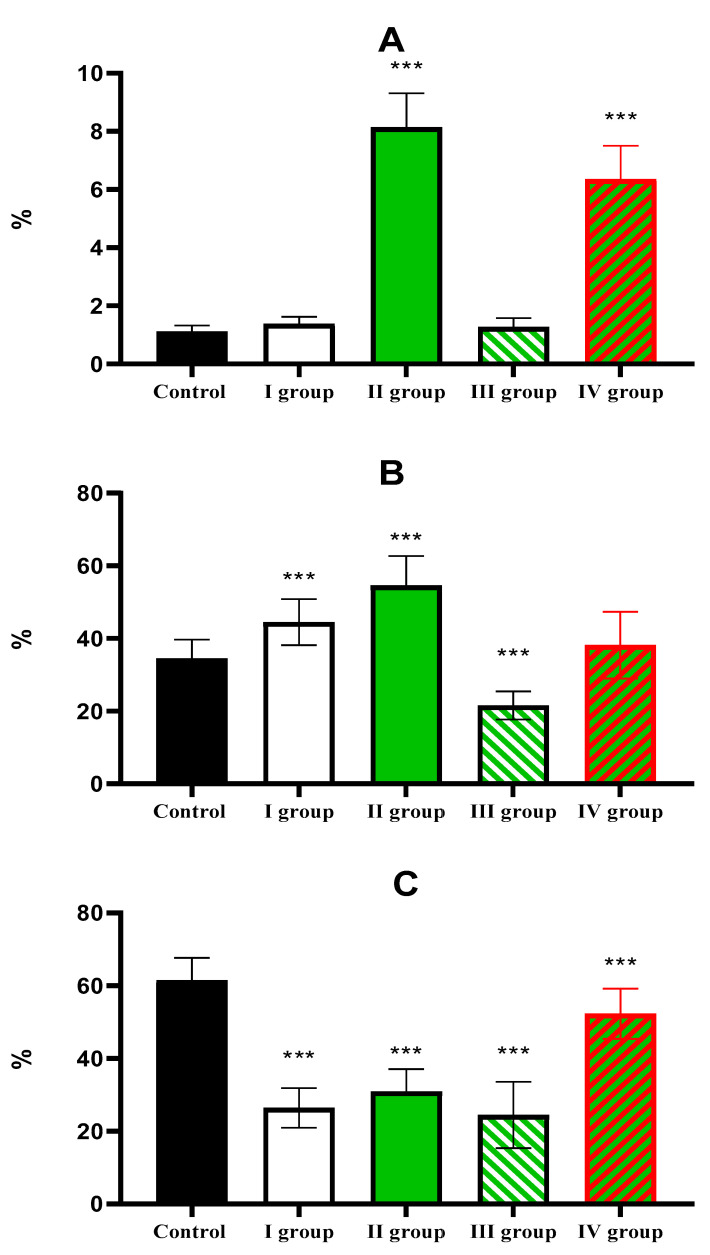
The individual and combined effects of dark deprivation, CCl_4_, and exogenous melatonin on the expression of (**A**) *Ki-67*; (**B**) *Per2*; (**C**) *Bmal1*. Note: hereinafter, *** *p* ≤ 0.0005 vs. control group.

Normally, *Bmal1* expression is observed in 61.51 ± 6.14% of hepatocytes; a decrease was observed in the livers of animals of experimental groups to 26.44 ± 5.45%, 30.96 ± 6.14%, and 24.51 ± 9.13% in the first three groups. The values in Group IV, amounting to 52.33 ± 6.88%, are lower than control values but higher than those in other treatment groups (Figure 9 and Figure 10).

### 3.4. Biochemical Studies

**Figure 11:**
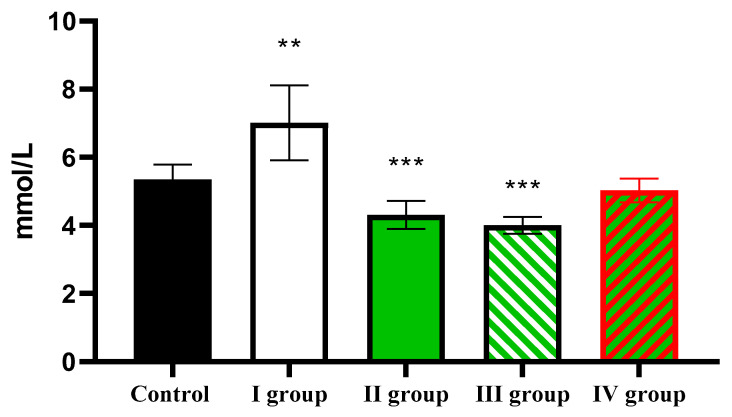
The individual and combined effects of dark deprivation, CCl_4_, and exogenous melatonin on blood glucose levels in rats. Note: hereinafter, ** *p* ≤ 0.005, *** *p* ≤ 0.0005 vs. control group.

Animals in the control group exhibited a blood glucose level of 5.35 ± 0.44 mmol/L. Dark deprivation leads to a rise in this indicator to 7.01 ± 1.10 mmol/L, but under the influence of CCl_4_, it decreases to 4.31 ± 0.41 mmol/L. The effect of dark deprivation together with CCl_4_ causes a further decrease in glucose level to 4.0 ± 0.25 mmol/L, but the use of melatonin causes the normalization of this indicator, which in rats of Group IV is 5.03 ± 0.35 mmol/L (Figure 11).

**Figure 12:**
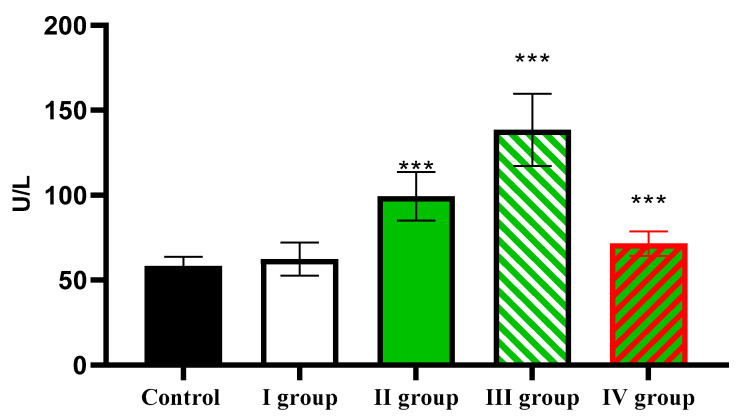
The individual and combined effects of dark deprivation, CCl_4_, and exogenous melatonin on ALT activity in the blood of rats. Note: hereinafter, *** *p* ≤ 0.0005 vs. control group.

Analysis of ALT activity showed that in the control, the activity of this transaminase was 58.46 ± 5.37 U/L; under conditions of dark deprivation it increased slightly; under the influence of CTC the ALT activity it increased to 99.35 ± 14.22 U/L; the effect of dark deprivation together with CCl_4_ caused a further increase in the indicator to 138.54 ± 21.25 U/L; and in rats of Group IV, it decreased to 71.51 ± 7.88 U/L, which remains reliably higher than the control (Figure 12).

**Figure 13:**
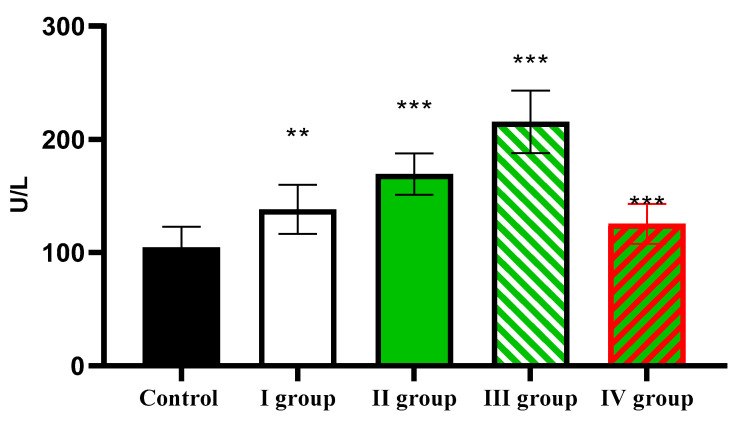
The individual and combined effects of dark deprivation, CCl_4_, and exogenous melatonin on AST activity in the blood of rats. Note: hereinafter, ** *p* ≤ 0.005, *** *p* ≤ 0.0005 vs. control group.

The AST activity level in the control was 104.51 ± 18.33 U/L, increasing in the first three experimental groups to 138.21 ± 21.55 U/L, 169.35 ± 18.33 U/L, and 215.51 ± 27.51 U/L, respectively. In animals of the last group, activity of AST was considerably lower than in rats of the experimental groups, but higher than in the control, and amounted to 125.56 ± 17.64 U/L (Figure 13).

**Figure 14:**
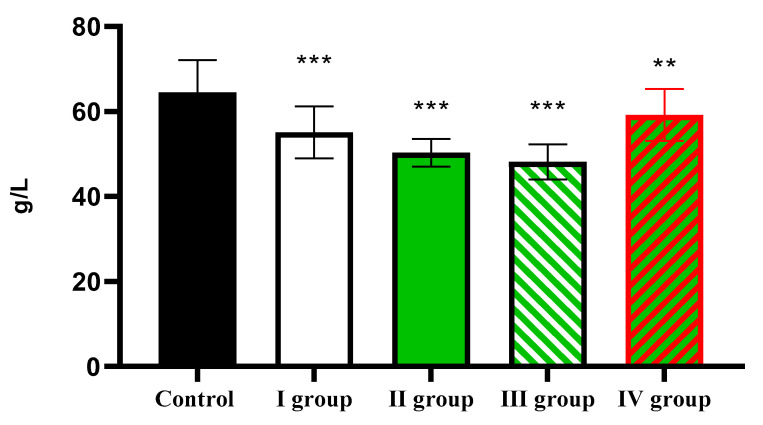
The individual and combined effects of dark deprivation, CCl_4_, and exogenous melatonin on the level of total protein in the blood of rats. Note: hereinafter, ** *p* ≤ 0.005, *** *p* ≤ 0.0005 vs. control group.

The level of total protein decreases from 64.51 ± 7.56 g/L in the control to 55.12 ± 6.14 g/L in Group I, 50.31 ± 3.28 g/L in Group II, 48.17 ± 4.16 g/L in Group III, and 59.17 ± 6.10 g/L in Group IV (Figure 14).

**Figure 15:**
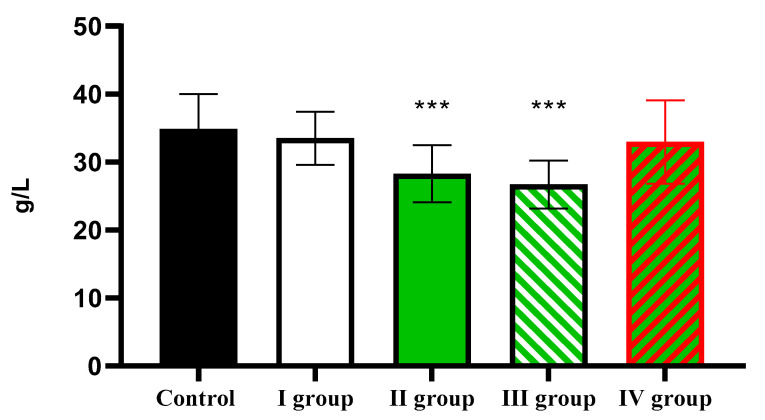
The individual and combined effects of dark deprivation, CCl_4_, and exogenous melatonin on the level of albumin in the blood of rats. Note: hereinafter, *** *p* ≤ 0.0005 vs. control group.

At the same time, the albumin level, which was 34.91 ± 5.11 g/L in the control, showed a significant decrease in Group II and Group III to 28.29 ± 2.17 g/L and 26.71 ± 6.51 g/L, respectively (Figure 15).

Thus, dark deprivation and CCl_4_ synergistically enhance liver damage. CCl_4_ causes fatty degeneration, necrosis, and inflammation, which are exacerbated by desynchronosis. Exogenous melatonin has a protective effect: it reduces necrosis and steatosis, partially restores circadian genes (Per2 and BMAL1), and normalizes biochemical parameters (ALT, AST, and glucose). Disruption of normal light patterns increases the liver’s sensitivity to toxins.

### 3.5. Analysis of Circadian Rhythmicity of the Studied Parameters

Cosinor analysis of the control group revealed a significant circadian rhythm in melatonin concentration, characterized by an acrophase at 01:24 and an amplitude of 13.94 pg/mL. Under conditions of dark deprivation, either alone or in combination with CCl_4_, no melatonin rhythm was observed. In Group II rats, the rhythm had an acrophase at 1:04 and an amplitude of 10.06 pg/mL. In contrast, Group IV exhibited an acrophase shifted to 2:24 and a reduced amplitude of 6.38 pg/mL.

Cosinor analysis revealed a reliable circadian rhythm in hepatocyte nuclear area in all groups except Group III. The daily dynamics of the cross-sectional hepatocyte area demonstrate a pronounced maximum noted at 9:00 and a minimum noted at 3:00. In Group I rats, the maximum was phase-shifted to 15:00 while the minimum remained unchanged. In rats of Group II and Group III, the daily dynamics of the hepatocyte cross-sectional area are practically not expressed, while in animals of Group IV, with a minimum at 15:00, the maximal value occurred at 3:00. Cosinor analysis of hepatocyte size daily dynamics revealed a disrupted rhythm in Groups II and III. Furthermore, the NCR rhythm was disrupted only in Group III (Table 3).

A circadian rhythm of *Ki-67* expression was observed in hepatocytes of animals in all groups except Group III, according to Cosinor analysis; however, the amplitude–phase characteristics of the rhythms differ significantly.

While cosinor analysis showed the disruption of *Per2* expression of circadian rhythms in Groups II and III, the rhythm parameters in Group IV remained comparable to controls.

The *Bmal1* rhythm was disrupted in Group III according to cosinor analysis. In contrast, Group IV exhibited rhythm parameters nearly identical to the control group (Table 4).

Analysis of circadian dynamics revealed significant rhythms in all studied biochemical parameters in control animals. For example, cosinor analysis revealed a significant circadian rhythm for glucose in the control group, as well as in Groups I and IV. The rhythms of the latter groups are characterized by a similar acrophase, occurring in the evening hours, and minor differences in the rhythm amplitude (Table 5).

CCl_4_ disrupts the CR of ALT, and the rhythms of rats of the control group and Groups I and IV occur in the evening hours but differ in amplitude. As with ALT, the AST rhythms in rats of Groups II and III are disrupted, and the acrophases of the remaining rhythms occur in the morning hours (Table 5).

The acrophase of the total protein rhythm occurred in the evening in control animals. While the rhythm was disrupted in Groups II and III, Groups I and IV exhibited a phase shift, with the acrophase advancing to the morning hours.

Consistent with the total protein rhythm, albumin exhibited an evening acrophase in both the control group and Group IV. Under constant illumination, the acrophase was shifted to the nighttime hours, and in rats of Groups II and III, the rhythm was disrupted (Table 5).

Thus, toxic damage and dark deprivation disrupt circadian rhythms of biochemical parameters, and melatonin exerts a protective effect. Dark deprivation and CCl_4_ cause a decrease in melatonin levels, disruption of circadian rhythms of morphological parameters and hepatocyte metabolism, dysregulation of clock genes (*Per2* and *Bmal1*), and a loss of diurnal oscillations in the studied biochemical parameters. Melatonin partially restores the rhythm of melatonin synthesis, circadian oscillations of metabolism (lipids, glycogen), the rhythms of *Per2* and *Bmal1* gene expression, and restores the normal dynamics of ALT, AST, glucose, and proteins.

## 4. Discussion

This study demonstrates the combined negative effects of dark deprivation (constant light) and the hepatotoxin CCl_4_ on the morphological and functional condition and circadian organization of liver of rats. The key finding of our study is the evidence that these two factors act synergistically, significantly increasing liver damage and disrupting its circadian rhythm. A key pathogenetic link in this process is the light-induced deficiency of endogenous melatonin, which is confirmed by the pronounced hepatoprotective and chronotropic (rhythm-restoring) effects of exogenous melatonin.

The data obtained clearly correlate the degree of liver damage with melatonin levels. Animals subjected to dark deprivation (Group I) showed a significant decrease in blood melatonin concentrations, accompanied by a moderate increase in damage markers (ALT and AST), the development of steatosis, and necrosis. This is consistent with the known antioxidant and antiapoptotic properties of melatonin [19,20]. CCl_4_, as a single factor (Group II), also reduced melatonin levels, likely due to general stress and impaired liver function, but the damage was more pronounced due to its direct toxic effect. The most severe damage, including massive centrilobular necrosis, critical levels of steatosis, and complete suppression of regenerative potential (minimal binucleated hepatocyte counts and *Ki-67* rhythm disturbances), was observed in Group III (CCl_4_ + dark deprivation). This indicates that melatonin deficiency caused by constant light exposure deprives the liver of its key endogenous defense mechanism, making it extremely vulnerable to toxins. Thus, melatonin is not simply a modulator, but a necessary factor in liver resistance.

Administration of exogenous melatonin (Group IV) completely reversed the hormone deficiency caused by dark deprivation and exerted a pronounced protective effect. This was demonstrated by a significant reduction in all pathomorphological parameters (NAS, steatosis, and necrosis) compared to Group III, as well as by the normalization of hepatocyte micromorphometric parameters (nuclear and cell area, NCA). Importantly, melatonin not only protected the parenchyma but also stimulated regenerative processes, as evidenced by the highest proportion of binucleated hepatocytes among all groups and increased Ki-67 expression.

Biochemical data confirm the morphological results: melatonin administration led to a significant reduction in ALT and AST activity and normalization of glucose, total protein, and albumin levels. This indicates restoration of the liver’s synthetic and metabolic functions.

Our study revealed that both dark deprivation and CCl_4_ intoxication individually disrupt circadian rhythms of various parameters, while their combined effect leads to almost complete desynchronosis.

It was expected that constant illumination (Groups I and III) would completely suppress melatonin’s circadian secretory rhythm, which represents a direct consequence of the suppression of pineal gland function.

The disruption of key clock gene expression is a central mechanism of desynchronosis. CCl_4_ intoxication (Group II) induced hyperexpression of *Per2* and suppression of *Bmal1*, which may reflect a cellular stress response. Combined treatment (Group III) resulted in complete disruption of both gene rhythms. Exogenous melatonin (Group IV) effectively restored normal expression and circadian rhythms of *Per2* and *Bmal1*, confirming its role as a potent chronobiotic.

Circadian rhythms of hepatocyte size and nuclei, as well as key biochemical parameters (glucose, ALT, AST, and proteins), were disrupted or completely destroyed in the toxic injury groups (Groups II and III). Melatonin in Group IV demonstrated the ability to restore or significantly improve the amplitude–phase characteristics of these rhythms, bringing them closer to control values.

This effect of melatonin can be explained by its ability to activate key regulatory pathways (such as SIRT1 and Nrf2) and directly influence the expression of circadian cycle genes through melatonin receptors in hepatocytes [31,32,33,34,35,36,37].

Limitations of this study: The main limitation is the animal model, which requires caution when extrapolating the results to humans. Furthermore, primarily short-term effects were studied; the long-term impact of melatonin rhythm correction on fibrosis or carcinogenesis in damaged liver requires further study.

## 5. Conclusions

Dark deprivation (constant light exposure) and the hepatotoxic effects of carbon tetrachloride act synergistically, potentiating structural and functional damage to the liver, manifested by increased steatosis, necrosis, impaired regenerative potential, and suppression of the organ’s synthetic function.

The key pathogenetic mechanism mediating the increased toxic effect of CCl_4_ during dark deprivation is a light-pollution-induced deficiency of endogenous pineal melatonin, a hormone with potent antioxidant and hepatoprotective properties.

The administration of exogenous melatonin provided significant hepatoprotection against the combined effects of dark deprivation and CCl_4_. It markedly attenuated necrosis and steatosis and restored normal levels of biochemical markers (ALT, AST, glucose, protein).

Both dark deprivation and CCl_4_ intoxication lead to profound disruptions in the liver’s circadian organization, manifested by desynchronization of clock gene expression (Per2, BMAL1), loss of circadian rhythms in hepatocyte morphometry, and key biochemical parameters.

Melatonin exhibits pronounced chronobiotic activity by effectively restoring circadian rhythms. It normalizes both the expression and its rhythmicity of *Per2* and *Bmal1* genes, and also the diurnal variations in key morphofunctional parameters of liver. This underscores its therapeutic potential not only as a hepatoprotector but also as a means of correcting desynchronosis in liver pathologies.

These data confirm the importance of considering circadian rhythms and factors that disrupt them (such as light pollution) when assessing the mechanisms of toxic liver damage and developing liver protection strategies.

## Figures and Tables

**Table 1:** Some pathomorphological indices of rat liver.

Group	NAS Index	Proportion of Hepatocytes Containing Lipid Droplets,%	Proportion of Necrotic Hepatocytes,%	Proportion of Binuclear Hepatocytes,%
Control (n = 40)	1.71 ± 0.21	1.70 ± 0.01	0.5 ± 0.04	7.04 ± 1.98
Group I (n = 40)	23.28 ± 4.22 ***	23.41 ± 3.22 ***	4.87 ± 0.57 ***	9.97 ± 1.60 *
Group II (n = 40)	33.40 ± 4.24 ***	37.60 ± 6.90 ***	35.82 ± 4.66 ***	5.14 ± 1.44 *
Group III (n = 40)	76.60 ± 6.90 ***	57.85 ± 12.14 ***	54.17 ± 9.13 ***	2.17 ± 0.21 **
Group IV (n = 40)	17.15 ± 2.17 ***	14.25 ± 6.67 ***	16.35 ± 6.17 ***	13.57 ± 0.81 ***

* *p* ≤ 0.05, ** *p* ≤ 0.005, *** *p* ≤ 0.0005.

**Table 2:** Impact of isolated and combined exposure to dark deprivation, CCl_4_, and exogenous melatonin on hepatocyte micromorphometry in rats.

	Cross-Sectional Area of Nuclei, µm^2^	Cell Cross-Sectional Area, µm^2^	NCR
Control, n = 40	41.72 ± 7.24	186.50 ± 28.51	0.22 ± 0.05
Group I, n = 40	44.55 ± 6.24	231.45 ± 23.01 ***	0.19 ± 0.02 **
Group II, n = 40	38.10 ± 3.25 *	201.10 ± 8.50 **	0.19 ± 0.04 **
Group III, n = 40	35.81 ± 3.05 ***	215.20 ± 17.18 ***	0.16 ± 0.03 ***
Group IV, n = 40	42.33 ± 6.25	196.35 ± 18.24	0.21 ± 0.05

* *p* ≤ 0.05, ** *p* ≤ 0.005, *** *p* ≤ 0.0005.

**Table 3:** Cosinor analysis of hepatocyte micromorphometric parameters.

Group	Parameter					
	Cross-Sectional Area of the Hepatocyte Nucleus, µm^2^		Hepatocyte Cross-Sectional Area, µm^2^		NCR	
	Amplitude	Acrophase	Amplitude	Acrophase	Amplitude	Acrophase
Control	11.06	14:08	31.57	11:48	0.032	16:18
Group I	7.58	12:54	29.37	15:54	0.024	10:08
Group II	3.66	18:15	No significant CR		0.019	18:24
Group III	No significant CR		No significant CR		No significant CR	
Group IV	7.63	19:12	20.31	14:06	0.046	21:54

**Table 4:** Cosinor analysis of circadian expression of studied proteins in hepatocytes.

Group	Parameter					
	Ki-67, %		Per2, %		BMAL1, %	
	Amplitude	Acrophase	Amplitude	Acrophase	Amplitude	Acrophase
Control	0.32	3:49	7.66	2:20	6.06	12:13
Group I	0.55	13:35	6.86	14:09	6.50	8:14
Group II	0.04	7:56	No significant CR		3.95	20:30
Group III	No significant CR		No significant CR		No significant CR	
Group IV	1.57	5:03	12.41	1:55	4.25	12:09

**Table 5:** Cosinor analysis of the circadian dynamics of plasma biomarkers in rats.

Group	Parameter								
	Glucose, mol/L			ALT, U/L		AST, U/L			
	Amplitude	Acrophase		Amplitude	Acrophase	Amplitude			Acrophase
Control	0.356	17:26		7.74	15:42	21.54			6:28
Group I	1.762	8:23		11.84	17:58	26.85			8:16
Group II	0.194	7:57		No significant CR		No significant CR			
Group III	No significant CR			No significant CR		No significant CR			
Group IV	0.427	16:12		8.55	15:40	19.81			8:21
	**Parameter**								
	**Total protein, g/L**				**Albumin, g/L**				
	**Amplitude**		**Acrophase**		**Amplitude**		**Acrophase**		
Control	3.85		16:56		7.59			15:58	
Group I	4.15		6:16		5.08			2:28	
Group II	No significant CR				No significant CR				
Group III	No significant CR				No significant CR				
Group IV	3.30		5:43		7.37			15:59	

## Data Availability

The original contributions presented in this study are included in the article. Further inquiries can be directed to the corresponding author.
